# Risk and Protective Factors for Injury in Adult Front- and Rear-Seated Motor Vehicle Occupants in New York State

**DOI:** 10.3390/ijerph21060663

**Published:** 2024-05-22

**Authors:** Laura Zhang, Emilia Pawlowski, Leah M. Hines, Michael J. Bauer, Joyce C. Pressley

**Affiliations:** 1 New York State Department of Health, Bureau of Occupational Health and Injury Prevention, Empire State Plaza, Corning Tower, Albany, NY 12237, USA; lz2722@caa.columbia.edu (L.Z.); emilia.pawlowski@health.ny.gov (E.P.); leah.hines@health.ny.gov (L.M.H.); michael.bauer@health.ny.gov (M.J.B.); 2 Departments of Epidemiology, Health Policy and Management, Columbia University, New York, NY 10032, USA

**Keywords:** restraint use, motor vehicle crash, injury severity, rear-seated adults

## Abstract

Although seatbelt use is known to reduce motor vehicle occupant crash injury and death, rear-seated adult occupants are less likely to use restraints. This study examines risk and protective factors associated with injury severity in front- and rear-seated adults involved in a motor vehicle crash in New York State. The Crash Outcome Data Evaluation System (CODES) (2016–2017) was used to examine injury severity in front- and rear-seated occupants aged 18 years or older (*N* = 958,704) involved in a motor vehicle crash. CODES uses probabilistic linkage of New York State hospitalization, emergency department, and police and motorist crash reports. Multivariable logistic regression models with MI analyze employed SAS 9.4. Odds ratios are reported as OR with 95% CI. The mortality rate was approximately 1.5 times higher for rear-seated than front-seated occupants (136.60 vs. 92.45 per 100,000), with rear-seated occupants more frequently unrestrained than front-seated occupants (15.28% vs. 1.70%, *p* < 0.0001). In adjusted analyses that did not include restraint status, serious injury/death was higher in rear-seated compared to front-seated occupants (OR:1.272, 1.146–1.412), but lower once restraint use was added (OR: 0.851, 0.771–0.939). Unrestrained rear-seated occupants exhibited higher serious injury/death than restrained front-seated occupants. Unrestrained teens aged 18–19 years old exhibit mortality per 100,000 occupants that is more similar to that of the oldest two age groups than to other young and middle-aged adults. Speeding, a drinking driver, and older vehicles were among the independent predictors of serious injury/death. Unrestrained rear-seated adult occupants exhibit higher severe injury/death than restrained front-seated occupants. When restrained, rear-seated occupants are less likely to be seriously injured than restrained front-seated occupants.

## 1. Introduction

Motor vehicle crashes are the third leading cause of injury deaths in adults aged 18 to 85 years of age in the United States (U.S.) [1]. While restraint use has been shown to reduce motor vehicle crash injury and mortality by 50%, many teens and adults continue to travel unrestrained [2,3,4]. Restraint laws are stronger and restraint use higher for infants and children than for adults traveling in the rear-seat [5,6,7]. At the time of this study, fewer than twenty U.S. states had a primary enforced rear-seat restraint law that covered all ages [8]. Restraint use is lower in adult rear-seated occupants and unrestrained adult rear-seated passengers have higher mortality and experience higher medical charges than their restrained counterparts [3,9].

Legislation in most states does not require rear-seated teen and adult populations to ride restrained [8,9]. At the time this study was performed, New York State (NYS) allowed passengers aged 16 years and older to ride legally unrestrained in the rear seat unless traveling with a junior licensed driver [5,9]. Much of the scientific literature on rear-seat injury has focused on mortality or has included infants, children, and/or adolescents in their study populations [6,7,10,11]. Reports of child passengers have demonstrated increased injury requiring emergency care, including traumatic brain injury (TBI) and facial injury, in unrestrained compared to restrained rear-seated passengers [6,7].

Several factors likely contribute to anecdotal reports that the rear-seat mortality advantage has been evaporating for adult occupants [11,12]. In addition to lagging restraint use laws for rear-seated adults, newly engineered front-seat safety measures preceded their introduction and widespread availability in the rear-seat [3,11,12]. Multi-generational and multi-positioned airbags and improvements in the driver’s and the front-seated occupant cage were introduced prior to those for rear-seated passengers.

While there is overwhelming evidence for infant and child occupants with regard to rear-seating restraint use, there is less information available for adult occupant crash type and injury severity across later models of the vehicle fleet. Updated research examining components of the safe system approach may allow for the identification of risk and protective factors associated with injury severity in rear-seated adults [13]. The aim of this study is to further our understanding of injury severity in rear-seated compared to front-seated adult occupants aged 18 years or older involved in a motor vehicle crash. Specifically, our objectives are (1) to characterize driver, vehicle, environmental, and crash characteristics by injury severity stratified by seating position and restraint status; and (2) to model independent predictors of death/severe injury vs. less severe/uninjured occupants in rear-seated compared to front-seated occupants. 

## 2. Materials and Methods

### 2.1. Data Source(s)

The Crash Outcome Data Evaluation System (CODES) from years 2016–2017 was used to identify restraint use, injury, vehicle, and crash characteristics for adult front- and rear-seated occupants. CODES links police and motorist crash records to hospitalization and emergency department records using a probabilistic linkage [14]. The probabilistic linkage was performed with LinkSolv Software 9.1.1473 (Strategic Matching, Inc., Morrisonville, NY, USA) [15]. CODES incorporates data from the NYS Department of Motor Vehicle police and motor vehicle crash reports, and discharge records received from hospital inpatient and emergency care [6,7,16]. The final analytic dataset used was de-identified and received clearance for use from the NYS Department of Health.

### 2.2. Study Population

The study population included front- and rear-seated (left-rear, middle-rear, and right-rear) passengers aged 18 years or older (*N* = 958,704) seated in a 4-wheel passenger vehicle involved in a crash in NYS between 2016 and 2017.

### 2.3. Exposure Variables

***Seating position.*** Seating position for rear-seated passenger occupants at the time of the crash was categorized into front-seated (*N* = 909,654) and rear-seated (*N* = 49,050). Unknown seating position (*N* = 560,396) for passenger occupants was excluded from the analysis.

***Restraint status.*** Restraint status was dichotomized into restrained or unrestrained for all adult occupants. 

### 2.4. Outcome Variables 

***Pre-hospital injury severity (KABCO).*** Occupant injury severity was examined using the KABCO score at the time of the crash [17]. The KABCO score categorized injury as killed, severe injury, moderate injury, minor injury, and uninjured. The KABCO score categories were collapsed into the following categories: killed, severe/moderate injury, and minor/uninjured.

***Traumatic brain injury* (TBI).** TBI was dichotomized as a yes/no variable using the International Classification of Diseases, 10th Revision, Clinical Modification (ICD-10-CM) codes and included S021, S028, S0291, S0402, S043, S0404, S06, S071, T744, S020 [18].

### 2.5. Covariate Variable Definitions

#### 2.5.1. Occupant Characteristics

***Occupant age.*** Occupant age was categorized into 18–19, 20–44, 45–64, 65–74, and 75 years and older. 

***Gender.*** Sex/gender for drivers and occupants was dichotomized as male or female.

***Number of passenger occupants.*** The number of rear-seated passengers riding in the vehicle at the time of the crash was categorized as 1, 2, 3, and 4 or more.

***Ejection status.*** The ejection status of vehicle occupants was categorized into (1) fully ejected; (2) partially ejected; and (3) not ejected.

#### 2.5.2. Vehicle Characteristics

***Vehicle year.*** The year of the vehicle make was categorized as: (1) <1999; (2) 1999 to 2003; (3) 2004 to 2008; (4) 2009 to 2014; and (5) 2015 to 2018 [19]. 

#### 2.5.3. Crash Characteristics

***Number of vehicles involved in crash.*** Crashes were categorized dichotomously as to whether they were single vehicle (one vehicle crash with no other vehicles involved) or multi-vehicle crashes (more than one vehicle involved in the crash). 

***Alcohol and/or drug involved crash.*** Alcohol and drug involvement in the crash was categorized as a yes/no variable.

***Speed-related crash.*** Speeding at the time of the crash was dichotomized into a yes/no variable.

***Air bag deployed.*** Airbag deployment at the time of the crash was categorized as a dichotomous yes/no variable.

***Crash time.*** Crash time was categorized as: (1) midnight–05:59 AM; (2) 06:00–09:59 AM; (3) 10:00 AM–3:59 PM; (4) 4:00–7:59 PM; and (5) 8:00–11:59 PM. 

***Lighting conditions.*** The lighting condition at the time of the crash was categorized into (1) daylight; (2) dawn; (3) dusk; (4) dark road, lighted; and (5) dark road, unlighted.

***Weather conditions.*** To assess the protentional association between weather conditions and injury, weather conditions were categorized into five categories: (1) clear; (2) cloudy; (3) rain; (4) snow; (5) sleet/hail; and (6) fog/smoke.

***Contributing factors.*** Crash-contributing factors were categorized into five categories (none, 1, 2, 3, 4 or more) that describe the number of outside factors that contributed to the vehicle’s crash.

***Type of collision.*** The type of collision was categorized as: (1) rear end; (2) overtaking; (3) left turn; (4) right turn/angle; (5) head on; (6) sideswipe; and (7) other.

***Vehicle movement before crash.*** Pre-collision movement of the vehicle was categorized as: (1) straight ahead; (2) turning; (3) accelerating; (4) decelerating/stopped; (5) parked; (6) changing lanes; (7) merging; (8) passing; (9) backing; and (10) other/unknown.

***Failure to yield right of way.*** Failure to yield at the time of the crash was dichotomized as a yes/no variable.

***Traffic control type.*** Traffic control type at the crash scene was categorized as: (1) traffic signal; (2) stop sign; (3) flashing light/yield sign; (4) no passing zone/RR crossing; (5) none; and (6) other/unknown.

***Disregarding traffic controls.*** Disregarding traffic controls at the time of the crash was dichotomized into a yes/no variable.

### 2.6. Statistical Analysis

The chi-square test statistic was used to examine bivariable associations between injury severity and potential covariates with a significance level defined as *p* ≤ 0.05. Variables were selected for investigation based on previously hypothesized or reported to be predictors of injury severity in front- and rear-seated adults. Mortality was calculated per 100,000 occupants by age group, seating position and restraint use. Covariates found to be significant at the 0.05 level were evaluated for inclusion in the final multilevel, multivariable logistic regression models [20]. A backward elimination approach was used in all regression models to select covariates for adjustment: we began with a saturated model, identified the covariate with the highest *p*-value, eliminated it, and refit the model. This process was repeated until all variables remaining in the equation were significant at *p* < 0.05. Statistical significance was determined by *p*-values < 0.05 and 95% confidence limits. Analyses were performed using Proc MI ANALYZE due to the imputed structure of the data, in SAS software version 9.4 (SAS Institute Inc., Cary, NC, USA). Unadjusted and adjusted odds ratios were reported with a 95% confidence interval. SAS 9.4 used to conduct all analyses [21,22].

## 3. Results

**Figure 1:**
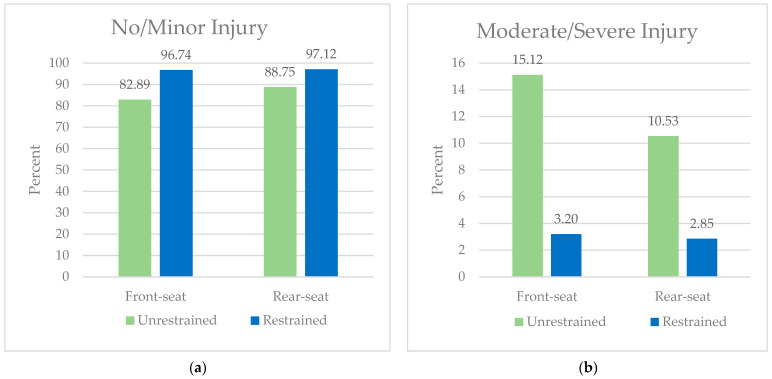

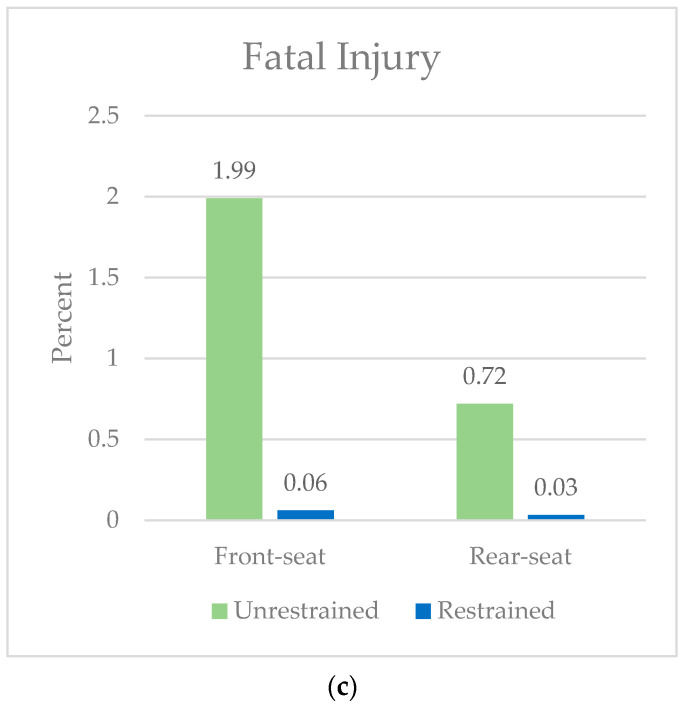
(**a**) Percent of front- and rear-seated occupants involved in a motor vehicle crash who had no or minor injury, stratified by restraint status. (**b**) Percent of front- and rear-seated occupants involved in a motor vehicle crash who had moderate or severe injury, stratified by restraint status. (**c**) Percent of front- and rear-seated occupants involved in a motor vehicle crash who sustained a fatal injury, stratified by restraint status.

The study population consisted of 958,704 front- and rear-seated occupants aged 18 years or older traveling in a four-wheeled passenger vehicle involved in a motor vehicle crash on a NYS roadway. Of these, 909,654 (94.88%) were front-seated and 49,050 (5.12%) were rear-seated. Occupants in the front seat were more likely to be restrained than occupants in the rear seat (98.30% vs. 84.72%, *p* < 0.001) (Table 1). Restrained occupants in the front seat and rear seat were less likely to experience injury compared to those who were similarly seated but unrestrained. The majority of front- and rear-seated occupants involved in a motor vehicle crash sustained minor or no injury (Figure 1a). The percent of unrestrained rear-seated occupants who experienced moderate-to-severe injury was 3.29 times higher than restrained front-seated occupants and 3.69 times higher than restrained rear-seated occupants (Figure 1b). The percent of unrestrained rear-seated occupant mortality was higher than the percent of restrained front-seated occupants (Figure 1c). The proportion of rear-seated restrained occupants who died was half that of front-seated restrained occupants (Figure 1c).

***Occupant age and sex.*** The study population was majority male (54.56%) and aged 20 to 44 years (Table 1). The majority of occupants experienced no injury/minor injury (Table 1). Across restraint status and front- and rear-seating positions, male mortality was double to triple the death of female occupants (Table 1). A higher portion of fatally injured front-seated occupants were male (77.53% vs. 22.48%, *p* < 0.0001) (Table 1). 

***Ejection status*.** Overall, 52.70% of fully ejected occupants were unrestrained. Nearly one third (32.19%) of unrestrained fatally injured occupants were partially or fully ejected from the vehicle. Among those fatally injured, a higher proportion of unrestrained occupants were either partially or fully ejected from the vehicle compared to restrained occupants (12.24% vs. 2.09%, *p* = 0.020) (Table 1).

### Crash and Vehicle Characteristics

***Single* vs. *multi-vehicle*.** Overall, 81.43% of crashes involved multiple vehicles (Table 2). For unrestrained front-seated occupants with moderate or severe injuries, about half were involved in single vehicle crashes (51.39%) (Table 2). When compared to restrained front-seated occupants, a higher portion of unrestrained front-seated occupants who died were involved in a single vehicle crash compared to a multi-vehicle crash (59.40% vs. 37.30%, *p* < 0.001). A higher proportion of restrained front-seated occupants died in multi-vehicle crashes compared to unrestrained front-seated occupants (62.80% vs. 37.20%, *p* < 0.001) (Table 2).

**Figure 2:**
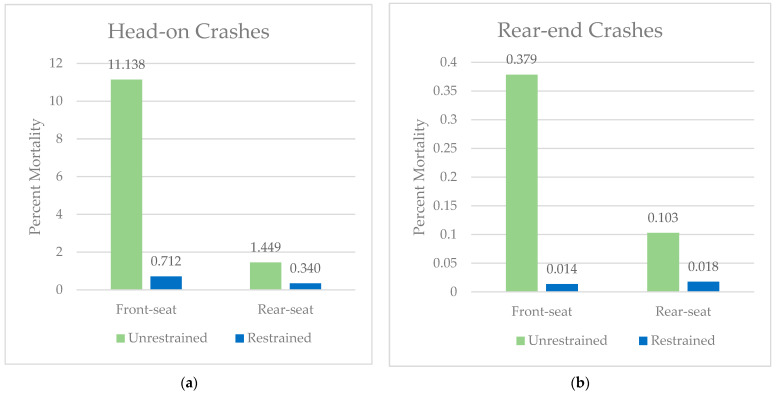
(**a**) Percent mortality of front- and rear-seated occupants involved in head-on crashes. (**b**) Percent mortality of front- and rear-seated occupants involved in rear-end crashes.

***Collision type.*** For front-seated restrained and unrestrained occupants, head-on crashes were more likely to be fatal compared to restrained and unrestrained rear-seated occupants (Figure 2a). Unrestrained front-seated occupants were more likely to die in a head-on crash compared to unrestrained rear-seated occupants (Figure 2a). For rear-end crashes, unrestrained front-seated occupants were more likely to die compared to unrestrained rear-seated occupants (Figure 2b).

***Alcohol-involved crash.*** Alcohol was involved in more than one-third of front-seat unrestrained deaths and in approximately 30% of unrestrained rear-seated deaths. Front-seated fatalities who were restrained had alcohol involvement that was approximately half that of unrestrained front-seated fatalities (34.23% vs. 17.13%, *p* < 0.001). In contrast, fewer than 2% of minor to no injury in restrained occupants were alcohol-involved crashes (Table 2). Compared to unrestrained occupants in the rear-seat, unrestrained occupants in the front-seat received more moderate to severe injuries if the driver was drinking or drugged (9.51% vs. 28.79%, *p* < 0.0001) (Table 2). 

***Time of day*.** Overall, approximately 36.09% of the crashes occurred between the hours of 10:00 AM and 4:00 PM (Table 2). A lower proportion of restrained front-seated occupants than rear-seated occupants were involved in crashes resulting in moderate or severe injury between the hours of 8:00 PM to 6:00 AM (23.99% vs. 36.26%) (Table 2). Among the unrestrained, a higher proportion of front-seated occupants compared to rear-seated occupants were involved in a fatal crash between the hours of 12:00 AM to 6:00 AM (22.82% vs. 31.48%, *p* < 0.001) (Table 2). A higher proportion of restrained front-seated occupants than rear-seated occupants died in crashes occurring between the hours of 8:00 PM and 12:00 AM (13.26% vs. 2.34%, *p* = 0.011) and 6:00 AM and 10:00 AM (14.55% vs. 7.69%, *p* = 0.012) (Table 2). 

***Number of passenger occupants.*** Approximately 84.37% of vehicles involved in collisions had one or two passenger occupants (Table 1). The majority (88.39%) of vehicles with front-seated occupants had one to two passenger occupants in the front seat, while the majority of vehicles with rear-seated passenger occupants had three or more passenger occupants (Table 1).

***Lighting conditions.*** Approximately 67.24% of crashes across restraint status and injury severity occurred during daylight hours (Table 2). Front-seated occupants who died (26.44%) were more likely to be in a crash on a dark unlit road than those who sustained no/minor injury (6.25%) or moderate/severe injury (17.43%) (Table 2). Both restrained and unrestrained rear-seated occupants were more likely to sustain moderate/severe injury on dark roads that were lighted compared to front-seat occupants. 

***Weather conditions.*** A higher proportion of restrained front-seated occupants than rear-seated occupants who died were involved in a crash during rainy weather (10.33% vs. 0.00%, *p* = 0.006) (Table 2). 

***Air bag deployment.*** Across restraint status and injury severity, air bags were more likely to be deployed for front-seated individuals compared to rear-seated individuals (Table 2).

***Yielding and disregarding traffic controls.*** Approximately 5% of crashes were due to disregarding traffic controls (Table 2). Unrestrained rear-seated occupants in a vehicle that failed to yield the right of way were more likely to have moderate or severe injuries compared to their front-seated counterparts (14.58% vs. 10.46%, *p* = 0.002) (Table 2). 

***Traffic control type*.** For both restrained and unrestrained, a higher proportion of front-seated than rear-seated occupants died in a crash in which there was no traffic control signal. (Restrained: 22.14% vs. 15.38%; Unrestrained: 25.00% vs. 14.81%). (Table 2). 

***Speeding*.** A higher proportion of crash deaths involved speeding than those that resulted in less severe injury (Table 2). Unrestrained front-seated occupants were more likely to die than restrained front-seated occupants if the vehicle was speeding (37.58% vs. 25.78%, *p* < 0.001). Among the unrestrained front-seated occupants were more likely than rear-seated occupants to be involved in crashes resulting in moderate or severe injury if the vehicle was speeding (30.91% vs. 20.03%, *p* < 0.001). (Table 2).

***Pre-collision movement*.** Across injury severity, restraint status, and seating position, the majority of vehicles were moving straight ahead before the collision occurred (Table 2).

***Vehicle model year.*** A higher proportion of rear-seated than front-seated occupants had a fatal collision in newer vehicle models manufactured between the years 2015 and 2018 (Restrained: 25.00% vs. 11.26%; Unrestrained: 21.15% vs. 9.47%,) (Table 2). A higher proportion of unrestrained front-seated occupants traveling in vehicle models made between the years of 1999 and 2003 were involved in fatal crashes compared to unrestrained rear-seated occupants (Table 2).

***Injury severity by collision manner.*** Rear-seated occupants who experienced moderate or severe injuries were more frequently involved in rear-end collisions or a right-turn collisions than front-seated occupants (Table 2). In addition, a higher proportion of restrained rear-seated occupants died in rear-end collisions compared to front-seated occupants (15.38% vs. 6.11%) (Table 2). 

***Traumatic Brain Injury (TBI)*.** In unadjusted subgroup analyses that examined three categories of injury (no/minor injury, moderate/severe, died), for occupants involved in crashes resulting in moderate or severe injury, a higher proportion of unrestrained occupants had TBI compared to restrained occupants regardless of seating position (Front seat: 18,43% vs. 9.09%, Rear seat: 21.29% vs. 9.91%) (Table 1). Unrestrained rear-seated occupants involved in fatal crashes were more likely to have TBI than those front-seated (44.44% vs. 19.67%) (Table 1).

***Mortality by age group, seating position and restraint status.*** Mortality per 100,000 occupants is shown in Table 3. The mortality rate was approximately 1.5 times higher for rear-seated than front-seated occupants (136.60 vs. 92.45 per 100,000) with rear-seated occupants more frequently unrestrained than front-seated occupants (15.28% vs. 1.70%, *p* < 0.001). All restrained age groups demonstrated lower mortality than similarly aged unrestrained occupants that were both front- and rear-seated. Mortality was lowest in rear-seated restrained occupants for all age groups. Front-seated restrained occupants demonstrated the second lowest mortality rate. In all age groups, rear-seated unrestrained occupants demonstrated higher mortality than similarly aged front-seated restrained occupants. Among the unrestrained population, 18–19 year olds demonstrated higher mortality than the 20–44 year olds and 45–64 year olds. In the oldest age group, 75 years and older, mortality was higher than similarly seated younger populations. This age group exhibited approximately one-third lower mortality for rear-seated restrained compared to front-seated restrained occupants (Table 3).

***Independent predictors of mortality and severe injury***. In adjusted analyses that did not include restraint status, serious injury/death was higher in rear- compared to front-seated occupants (OR: 1.272, 1.146–1.412), but lower once restraint use was added (OR: 0.804, 0.715–0.904) (Table 4). Independent predictors of severe injury or death in a multi-vehicle crash included being unrestrained, speeding, a drinking driver, older vehicle, and frontal collision. When controlling for restraint status, rear-seated occupants were less likely to die compared to front-seated (OR: 0.804, 0.715, 0.904) (Table 4). In the adjusted multivariable model not controlling for restraint status, sitting in the rear-seat was associated with a 20.8% increase in mortality or severe injury (Table 4). When not controlling for restraint status in the adjusted model, occupants who were fully ejected from the vehicle at the time of the crash were nearly 19 times more likely to experience mortality or severe injury (Table 4). Occupants seated in newer vehicle models were less likely to experience mortality or severe injury in both the adjusted model not controlling for restraint status and the adjusted model controlling for restraint status (Table 4).

## 4. Discussion

This study employs a dataset that includes both fatal and nonfatal crashes as well as injury outcomes, vehicle, crash, behavioral, and environmental factors to extend the science of adult rear-seat safety [6,7,9]. It demonstrates that the relative rear-seat vs. front-seat advantage has narrowed to the point where, as an adult, it is safer to be front-seated and restrained than to be rear-seated and unrestrained, thus further debunking a lingering widely held belief that adults can ride safely unrestrained in the rear seat. 

An unexpected finding is that unrestrained rear-seated teens aged 18–19 years have crash mortality rates that are more similar to the older adult age groups than to other young and middle-aged adults. Other studies have documented that speed, distracted driving, and thrill seeking are factors contributing to the increased crash risk observed in young novice drivers [5,10,13,23]. Further study is needed to assess contributing factors, including the role of speed, in the apparent increased severity of unrestrained teen crashes.

This study, performed prior to passage of the NYS near-universal restraint law, demonstrates that rear-seated adult passengers had more serious injury than front-seated passengers. This is documented to be due to differential restraint use. With the passage of an all-age restraint law in NY, this study could serve as a basis for a follow-up examination of the effectiveness of the law to improve crash outcomes for rear-seated adults involved in a motor vehicle crash. This study also provides information that could inform pre-hospital response personnel and emergency department staff who are charged with providing clinical care, management and stabilization of victims of road traffic crashes. The finding that front-seated occupants were more likely to be restrained than rear-seated occupants is consistent with previous research on restraint status and seating position [23,24].

Vehicle age showed a consistent dose response effect across vehicle model year with newer vehicles proving to be a protective factor against severe injury/fatality in both adjusted and unadjusted models. This finding, in both fatal and nonfatal crashes, is consistent with previous studies that report lower mortality with newer vehicle model years [3,19]. Erosion of the relative protective advantage of the rear seat is associated with several improved safety designs associated with lowered injury. Some of these safety features confer benefit both front- and rear-seat occupants, but many, such as three-point safety belts, were made available for front-seats years ahead of the rear-seat. Many of the vehicle design changes, such as strengthening of the driver’s occupant cage and airbags, were initially introduced as safety features for drivers before other front-seated passengers and later for all front-seat occupants before being made available for the rear seat. Other features, such as multiple airbags and second-generation airbags, were also made available, sometimes decades earlier for front- compared to rear-vehicle compartments. 

The introduction of engineering safety advances for the rear seat continue to lag those introduced for the front seat. While some legislatively mandated safety features, such as crumple zones, benefitted both front- and rear-seated occupants, many currently manufactured vehicles do not have any rear-seat airbags. Second and third generation airbags, such as side, head, and knee airbags introduced for front-seated occupants, improved protection and decreased occupant airbag-associated injury [19]. Many of these safety features may contribute to our findings that newer vehicles are protective against serious injury and that riding unrestrained in the rear seat is associated with higher mortality rates than riding restrained in the front seat.

In multivariable analysis before controlling for restraint status, rear-seated occupants were approximately one-fifth more likely to be severely injured or killed compared to front-seated occupants. However, after controlling for restraint status, the rear seat was observed to be more protective than the front seat for mortality/severe injury. 

Across restraint status in front-seated occupants, head-on collisions were more fatal than rear-end, sideswipe, and angled (left and right) collisions. It is not surprising that this study found TBI to be higher in both unrestrained front- and unrestrained rear-seated occupants. Of note is that TBI, as identified in hospitalization and emergency department records, was categorized by officers on the crash scene as having no or minor injury in more than three quarters of occupants subsequently receiving a TBI diagnosis. Although further study is needed to confirm and characterize the healthcare-rendered TBI diagnoses, many occupants sustained minor concussions which may not have been apparent on the scene. 

This study has limitations. We used the well-documented method of matching hospitalization and emergency department data with crash data. It is possible that some crashes were missed due to being transported to out-of-state hospitals or due to a failure of records in the two data sets to match. Although it was not possible to control for other factors known to be important in motor vehicle injury such as the striking/struck vehicle weight and size or the vehicle travel speeds at the time of the crash, these vehicle features are important predictors of injury severity in both front- and rear-seated occupants [3,4,19]. We used the KABCO score given by officers at the scene for the measure of injury severity. Injury severity scores calculated from ICD-10 cm codes were not available in the data set used at the time of this study.

## 5. Conclusions

In conclusion, rear-seated adult occupants are at greater risk of injury than front-seated occupants traveling on NYS highways. This finding is due to differences in restraint use, with rear-seated occupants being less likely to be restrained. Restrained rear-seated occupants are less likely to be seriously injured than unrestrained rear-seated occupants and are less likely to experience serious injury than front-seated occupants. This study was conducted with data from years before NYS extended their restraint law beyond the all-age front-seat law to require teen and adult rear-seated occupants of all ages to be restrained. Further study is needed to assess the legislative impact on injury in rear-seated teens and adults. 

## Figures and Tables

**Table 1:** Person-level characteristics for motor vehicle crashes involving vehicle occupants aged 18 or older in New York State stratified by injury severity, restraint status, and seating position at time of crash, New York State Outcome Evaluation System (CODES).

	No/Minor Injury				Moderate/Severe Injury				Fatal			
	Unrestrained		Restrained		Unrestrained		Restrained		Unrestrained		Restrained	
Person-Level Characteristics	Front Seat	Rear Seat	Front Seat	Rear Seat	Front Seat	Rear Seat	Front Seat	Rear Seat	Front Seat	Rear Seat	Front Seat	Rear Seat
	*n* (%)				*n* (%)				*n* (%)			
*N*	12,419	6652	865,506	40,358	2265	789	28,623	1184	298	54	543	13
Occupant Characteristics Age												
18–19	587 (4.74)	591 (9.03)	44,458 (5.16)	4585 (11.40)	161 (7.13)	80 (10.18)	1871 (6.58)	131 (11.15)	14 (4.73)	7 (12.96)	28 (5.25)	0 (0.00)
20–44	7187 (58.01)	4213 (64.36)	453,524 (52.65)	23,605 (58.69)	1383 (61.22)	499 (63.49)	15,004 (52.73)	665 (56.60)	147 (49.66)	32 (59.26)	180 (33.83)	4 (30.77)
45–64	3566 (28.78)	1347 (20.58)	261,929 (30.41)	8367 (20.80)	501 (22.18)	142 (18.07)	7392 (25.98)	249 (21.19)	73 (24.66)	6 (11.11)	146 (27.44)	1 (7.69)
65–74	747 (6.03)	286 (4.37)	67,185 (7.80)	2271 (5.65)	130 (5.75)	36 (4.58)	2310 (8.12)	71 (6.04)	26 (8.78)	4 (7.41)	66 (12.41)	5 (38.46)
75 and older	302 (2.44)	109 (1.67)	34,222 (3.97)	1394 (3.47)	84 (3.72)	29 (3.69)	1876 (6.59)	59 (5.02)	36 (12.16)	5 (9.26)	112 (21.05)	3 (23.08)
Gender												
Male	8642 (69.63)	3033 (46.34)	475,864 (54.99)	16,964 (42.10)	1612 (71.17)	376 (47.66)	15,510 (54.19)	480 (40.61)	231 (77.52)	36 (66.67)	348 (64.09)	8 (61.54)
Female	3770 (30.37)	3512 (53.66)	389,559 (45.01)	23,335 (57.90)	653 (28.83)	413 (52.34)	13,110 (45.81)	702 (59.39)	67 (22.48)	18 (33.33)	195 (35.91)	5 (38.46)
Number of Passenger Occupants												
1	7856 (64.09)	137 (2.09)	543,836 (62.99)	482 (1.20)	1541 (68.31)	17 (2.15)	19,076 (66.72)	23 (1.94)	213 (71.48)	0 (0.00)	328 (60.41)	0 (0.00)
2	2928 (23.89)	1380 (21.06)	218,768 (25.34)	4447 (11.03)	526 (23.32)	202 (25.60)	6713 (23.48)	195 (16.47)	64 (21.48)	4 (7.41)	158 (29.10)	0 (0.00)
3	889 (7.25)	2342 (35.74)	59,049 (6.84)	15,408 (38.21)	119 (5.27)	302 (38.28)	1764 (6.17)	459 (38.77)	11 (3.69)	20 (37.04)	38 (7.00)	9 (69.23)
4 or more	584 (4.75)	2694 (41.11)	41,721 (4.83)	19,988 (49.57)	70 (3.10)	268 (33.97)	1036 (3.62)	507 (42.82)	10 (3.36)	30 (55.56)	19 (3.50)	4 (30.77)
Occupant Ejection Status												
Fully ejected	76 (0.64)	15 (0.23)	265 (0.03)	12 (0.03)	149 (6.67)	48 (6.20)	39 (0.14)	1 (0.09)	51 (17.11)	23 (42.59)	8 (1.47)	0 (0.00)
Partially ejected	50 (0.42)	17 (0.26)	507 (0.06)	39 (0.10)	64 (2.86)	7 (0.90)	64 (0.23)	1 (0.09)	36 (12.08)	3 (5.56)	11 (2.03)	0 (0.00)
Not ejected	11,832 (98.95)	6469 (99.51)	851,802 (99.91)	39,876 (99.87)	2022 (90.47)	719 (92.89)	28,163 (99.64)	1166 (99.83)	210 (70.47)	28 (51.85)	524 (96.50)	13 (100.00)
Occupant TBI												
No	1807 (94.56)	915 (96.01)	91,557 (96.15)	4008 (95.93)	686 (81.57)	196 (78.71)	8757 (90.91)	309 (90.09)	49 (80.33)	5 (55.56)	109 (80.15)	1 (100.00)
Yes	104 (5.44)	38 (3.99)	3664 (3.85)	170 (4.07)	155 (18.43)	53 (21.29)	876 (9.09)	34 (9.91)	12 (19.67)	4 (44.44)	27 (19.85)	0 (0.00)

**Table 2:** Crash and vehicle-related characteristics for motor vehicle crashes involving vehicle occupants aged 18 or older in New York State stratified by injury severity, restraint status, and seating position, New York State CODES data.

	No/Minor Injury				Moderate/Severe Injury				Fatal			
Crash- and Vehicle-Level Characteristics	Unrestrained		Restrained		Unrestrained		Restrained		Unrestrained		Restrained	
	Front Seat	Rear Seat	Front Seat	Rear Seat	Front Seat	Rear Seat	Front Seat	Rear Seat	Front Seat	Rear Seat	Front Seat	Rear Seat
	*n* (%)				*n* (%)				*n* (%)			
*N*	12,419	6652	865,506	40,358	2265	789	28,623	1184	298	54	543	13
Crash Characteristics												
Vehicles Involved in Crash												
Single vehicle	3109 (25.03)	1035 (15.77)	156,469 (18.08)	6113 (15.15)	1164 (51.39)	258 (32.70)	9070 (31.69)	268 (22.64)	177 (59.40)	29 (53.70)	202 (37.20)	2 (15.38)
Multi-vehicle	9310 (74.97)	5527 (84.23)	709,037 (81.92)	34,245 (84.85)	1101 (48.61)	531 (67.30)	19,553 (68.31)	916 (77.36)	121 (40.60)	25 (46.30)	341 (62.80)	11 (84.62)
Air Bag Deployed												
No	8407 (70.64)	6264 (97.75)	816,427 (94.33)	39,711 (98.40)	1330 (58.98)	731 (93.60)	20,641 (72.12)	1093 (92.31)	106 (35.57)	43 (84.31)	192 (35.56)	10 (76.92)
Yes	3495 (29.36)	144 (2.25)	49,079 (5.67)	647 (1.60)	925 (41.02)	50 (6.40)	7980 (27.88)	91 (7.69)	192 (64.43)	8 (15.69)	351 (64.64)	3 (23.08)
Driver Drinking or Drugged												
No	11,578 (93.23)	6438 (98.11)	853,297 (98.59)	39,935 (98.95)	1613 (71.21)	714 (90.49)	26,405 (92.25)	1140 (96.28)	196 (65.77)	38 (70.37)	450 (82.87)	10 (76.92)
Yes	841 (6.77)	124 (1.89)	12,209 (1.41)	423 (1.05)	652 (28.79)	75 (9.51)	2218 (7.75)	44 (3.72)	102 (34.23)	16 (29.63)	93 (17.13)	3 (23.08)
Speeding												
No	11,207 (90.24)	6049 (92.18)	817,836 (94.49)	38,023 (94.21)	1565 (69.09)	631 (79.97)	24,332 (85.01)	996 (84.12)	186 (62.42)	32 (59.26)	403 (74.22)	11 (84.62)
Yes	1212 (9.76)	513 (7.82)	47,671 (5.51)	2335 (5.79)	700 (30.91)	158 (20.03)	4291 (14.99)	188 (15.88)	112 (37.58)	22 (40.74)	140 (25.78)	2 (15.38)
Collision Type												
Rear-end	2658 (22.86)	1801 (28.64)	239,605 (28.62)	11,115 (28.55)	237 (10.70)	143 (18.89)	4392 (15.73)	218 (19.12)	11 (3.73)	2 (3.70)	33 (6.11)	2 (15.38)
Overtaking	1236 (10.63)	835 (13.28)	89,897 (10.74)	5105 (13.11)	62 (2.80)	33 (4.36)	1139 (4.08)	57 (5.00)	4 (1.36)	4 (7.41)	8 (1.48)	0 (0.00)
Left-turn	668 (5.74)	408 (6.49)	56,602 (6.76)	2415 (6.20)	104 (4.70)	46 (6.08)	2088 (7.48)	88 (7.72)	6 (2.03)	0 (0.00)	24 (4.44)	1 (7.69)
Right Turn/Angle	1598 (13.74)	1032 (16.41)	140,829 (16.82)	6356 (16.33)	209 (9.44)	96 (12.68)	4703 (16.85)	225 (19.74)	21 (7.12)	5 (9.26)	77 (14.26)	2 (15.38)
Head-on	248 (2.13)	97 (1.54)	12,122 (1.45)	548 (1.41)	119 (5.37)	39 (5.15)	1547 (5.54)	39 (3.42)	46 (15.59)	2 (3.70)	98 (18.15)	2 (15.38)
Sideswipe	167 (1.44)	78 (1.24)	13,437 (1.61)	567 (1.46)	29 (1.31)	7 (0.92)	568 (2.03)	16 (1.40)	4 (1.36)	0 (0.00)	11 (2.04)	0 (0.00)
Other	5054 (43.46)	2037 (32.40)	284,619 (34.00)	12,819 (32.93)	1454 (65.67)	393 (51.92)	13,476 (48.28)	497 (43.60)	203 (68.81)	41 (75.93)	289 (53.52)	6 (46.15)
Disregarding Traffic Controls												
No	11,711 (94.30)	6097 (92.91)	825,213 (95.34)	38,156 (94.54)	2099 (92.67)	734 (93.03)	26,516 (92.64)	1079 (91.13)	286 (95.97)	50 (92.59)	502 (92.45)	13 (100.00)
Yes	708 (5.70)	565 (7.09)	40,293 (4.66)	2202 (5.46)	166 (7.33)	55 (6.97)	2107 (7.36)	105 (8.87)	12 (4.03)	4 (7.41)	41 (7.55)	0 (0.00)
Traffic Control Type												
Traffic signal	6454 (55.04)	3292 (50.57)	456,279 (54.11)	20,986 (52.80)	1389 (61.93)	410 (52.23)	15,750 (55.62)	607 (51.79)	175 (59.12)	38 (70.37)	302 (55.72)	9 (69.23)
Stop sign	3373 (28.77)	2029 (31.17)	219,335 (26.01)	11,014 (27.71)	352 (15.69)	220 (28.03)	5777 (20.40)	313 (26.71)	24 (8.11)	3 (5.56)	52 (9.59)	1 (7.69)
Flashing light/yield sign	1081 (9.22)	741 (11.38)	90,970 (10.79)	4389 (11.04)	185 (8.25)	61 (7.77)	3115 (11.00)	158 (13.48)	17 (5.74)	5 (9.26)	55 (10.15)	1 (7.69)
No passing zone/RR crossing	116 (0.99)	66 (1.01)	11,952 (1.42)	491 (1.24)	16 (0.71)	4 (0.51)	257 (0.91)	8 (0.68)	1 (0.34)	0 (0.00)	3 (0.55)	0 (0.00)
None	445 (3.79)	244 (3.75)	50,124 (5.94)	1746 (4.39)	268 (11.95)	71 (9.04)	3049 (10.77)	67 (5.72)	74 (25.00)	8 (14.81)	120 (22.14)	2 (15.38)
Other	257 (2.19)	138 (2.12)	14,534 (1.72)	1119 (2.82)	33 (1.47)	19 (2.42)	370 (1.31)	19 (1.62)	5 (1.69)	0 (0.00)	10 (1.85)	0 (0.00)
Failure to Yield Right of Way												
No	10,676 (85.97)	5570 (84.88)	711,670 (82.23)	33,999 (84.24)	2028 (89.54)	674 (85.42)	23,156 (80.90)	975 (82.35)	279 (93.62)	52 (96.30)	464 (85.45)	10 (76.92)
Yes	1743 (14.03)	992 (15.12)	153,836 (17.77)	6359 (15.76)	237 (10.46)	115 (14.58)	5467 (19.10)	209 (17.65)	19 (6.38)	2 (3.70)	79 (14.55)	3 (23.08)
Pre-collision Movement												
Straight ahead	7411 (59.94)	3825 (58.38)	475,139 (54.93)	23,076 (57.21)	1767 (78.74)	617 (78.20)	21,056 (73.65)	868 (73.37)	257 (86.53)	41 (75.93)	439 (81.00)	10 (76.92)
Turning	1630 (13.18)	749 (11.43)	113,586 (13.13)	4738 (11.75)	197 (8.78)	70 (8.87)	3160 (11.05)	125 (10.57)	12 (4.04)	2 (3.70)	44 (8.12)	1 (7.69)
Accelerating	233 (1.88)	104 (1.59)	20,256 (2.34)	732 (1.81)	20 (0.89)	8 (1.01)	302 (1.06)	7 (0.59)	N/A	N/A	5 (0.92)	1 (7.69)
Decelerating or Stopped	1300 (10.52)	1297 (19.80)	174,992 (20.23)	8603 (21.33)	48 (2.14)	45 (5.70)	2655 (9.29)	116 (9.81)	6 (2.02)	3 (5.56)	13 (2.40)	1 (7.69)
Parked	109 (0.88)	34 (0.52)	3376 (0.39)	78 (0.19)	12 (0.53)	1 (0.13)	55 (0.19)	0 (0.00)	N/A	N/A	N/A	N/A
Changing lanes	370 (2.99)	233 (3.56)	25,533 (2.95)	1379 (3.42)	62 (2.76)	20 (2.53)	605 (2.12)	31 (2.62)	6 (2.02)	4 (7.41)	13 (2.40)	0 (0.00)
Merging	109 (0.88)	72 (1.10)	7589 (0.88)	424 (1.05)	8 (0.36)	6 (0.76)	119 (0.42)	8 (0.68)	1 (0.34)	0 (0.00)	2 (0.37)	0 (0.00)
Passing	76 (0.61)	54 (0.82)	5297 (0.61)	243 (0.60)	22 (0.98)	6 (0.76)	173 (0.61)	9 (0.76)	6 (2.02)	0 (0.00)	8 (1.48)	0 (0.00)
Backing	368 (2.98)	66 (1.01)	22,054 (2.55)	516 (1.28)	25 (1.11)	1 (0.13)	91 (0.32)	1 (0.08)	3 (1.01)	0 (0.00)	1 (0.18)	0 (0.00)
Other or Unknown	757 (6.12)	118 (1.80)	17,201 (1.99)	548 (1.36)	83 (3.70)	15 (1.90)	373 (1.30)	18 (1.52)	6 (2.02)	4 (7.41)	17 (3.14)	0 (0.00)
Weather Conditions												
Clear	8194 (69.22)	4399 (67.47)	521,357 (61.50)	26,394 (66.16)	1382 (61.37)	523 (66.62)	17,189 (60.48)	752 (63.89)	164 (55.41)	35 (64.81)	280 (51.66)	7 (53.85)
Cloudy	2070 (17.49)	1232 (18.90)	202,317 (23.86)	7968 (19.97)	555 (24.64)	162 (20.64)	6983 (24.57)	242 (20.56)	84 (28.38)	12 (22.22)	169 (31.18)	5 (38.46)
Rain	1141 (9.64)	624 (9.57)	77,810 (9.18)	3663 (9.18)	207 (9.19)	72 (9.17)	2572 (9.05)	124 (10.54)	27 (9.12)	4 (7.41)	56 (10.33)	0 (0.00)
Snow	366 (3.09)	217 (3.33)	39,371 (4.64)	1535 (3.85)	82 (3.64)	20 (2.55)	1308 (4.60)	42 (3.57)	12 (4.05)	3 (5.56)	24 (4.43)	1 (7.69)
Sleet, hail, or freezing rain	37 (0.31)	32 (0.49)	5014 (0.59)	249 (0.62)	14 (0.62)	6 (0.76)	264 (0.93)	8 (0.68)	5 (1.69)	0 (0.00)	7 (1.29)	0 (0.00)
Fog, smog, or smoke	30 (0.25)	16 (0.25)	1892 (0.22)	86 (0.22)	12 (0.53)	2 (0.25)	106 (0.37)	9 (0.76)	4 (1.35)	0 (0.00)	6 (1.11)	0 (0.00)
Time of Day												
Midnight–5:59 AM	1622 (13.36)	978 (15.05)	49,898 (5.82)	3760 (9.40)	515 (22.82)	196 (25.00)	3268 (11.47)	210 (17.92)	68 (22.82)	17 (31.48)	89 (16.39)	3 (23.08)
6:00–9:59 AM	1817 (14.96)	787 (12.11)	146,455 (17.09)	4024 (10.06)	309 (13.69)	85 (10.84)	4499 (15.79)	126 (10.75)	48 (16.11)	6 (11.11)	79 (14.55)	1 (7.69)
10:00–3:59 PM	3746 (30.85)	1833 (28.21)	315,250 (36.80)	13,762 (34.41)	547 (24.24)	212 (27.04)	10,083 (35.38)	353 (30.12)	66 (22.15)	10 (18.52)	200 (36.83)	4 (30.77)
4:00–7:59 PM	3065 (25.24)	1601 (24.64)	250,778 (29.27)	11,372 (28.43)	471 (20.87)	160 (20.41)	7084 (24.86)	268 (22.87)	57 (19.13)	11 (20.37)	103 (18.97)	3 (23.08)
8:00–11:59 PM	1893 (15.59)	1298 (19.98)	94,377 (11.02)	7076 (17.69)	415 (18.39)	131 (16.71)	3567 (12.52)	215 (18.34)	59 (19.80)	10 (18.52)	72 (13.26)	2 (2.34)
Lighting Conditions												
Daylight	7061 (59.55)	3600 (55.16)	588,551 (69.36)	24,143 (60.46)	1107 (49.09)	384 (48.92)	18,700 (65.73)	635 (53.86)	125 (42.37)	21 (38.89)	330 (61.00)	8 (61.54)
Dawn	267 (2.25)	121 (1.85)	15,160 (1.79)	517 (1.29)	47 (2.08)	33 (4.20)	603 (2.12)	21 (1.78)	10 (3.39)	2 (3.70)	9 (1.66)	0 (0.00)
Dusk	422 (3.56)	197 (3.02)	26,313 (3.10)	1352 (3.39)	66 (2.93)	14 (1.78)	781 (2.75)	34 (2.88)	9 (3.05)	0 (0.00)	8 (1.48)	0 (0.00)
Dark-road lighted	3367 (28.39)	2249 (34.46)	150,370 (17.72)	10,770 (26.97)	642 (28.47)	271 (34.52)	5227 (18.37)	372 (31.55)	73 (24.75)	18 (33.33)	87 (16.08)	3 (23.08)
Dark-road unlighted	741 (6.25)	359 (5.50)	68,173 (8.03)	3148 (7.88)	393 (17.43)	83 (10.57)	3138 (11.03)	117 (9.92)	78 (26.44)	13 (24.07)	107 (19.78)	2 (15.38)
Vehicle Characteristics												
Vehicle Make Year												
<1999	424 (3.77)	155 (2.57)	29,679 (3.61)	922 (2.49)	204 (9.60)	29 (3.89)	1737 (6.35)	37 (3.39)	26 (9.12)	2 (3.85)	41 (7.69)	0 (0.00)
1999 to 2003	1416 (12.60)	593 (9.82)	106,131 (12.91)	4179 (11.28)	438 (20.62)	100 (13.42)	4686 (17.13)	142 (13.02)	75 (26.32)	6 (11.54)	102 (19.14)	2 (16.67)
2004 to 2008	2711 (24.12)	1393 (23.07)	205,180 (24.96)	8525 (23.02)	633 (29.80)	166 (22.28)	7297 (26.68)	252 (23.10)	89 (31.23)	17 (32.69)	187 (35.08)	3 (25.00)
2009 to 2014	4041 (35.96)	2363 (39.13)	296,886 (36.11)	13,556 (36.60)	565 (26.60)	266 (35.70)	8659 (31.66)	389 (35.66)	68 (23.86)	16 (30.77)	143 (26.38)	4 (33.33)
2015 to 2018	2646 (23.55)	1535 (25.42)	184,292 (22.42)	9857 (26.61)	284 (13.37)	184 (24.70)	4975 (18.19)	271 (24.84)	27 (9.47)	11 (21.15)	60 (11.26)	3 (25.00)

**Table 3:** Differential motor vehicle mortality (per 100,000) by age, restraint status, and seating position, NYS CODES 2016–2017. CODES, Crash Outcome Data Evaluation System.

Age (yrs)	Front Restrained	Front Unrestrained	Rear Restrained	Rear Unrestrained	Total Restrained and Unrestrained
18–19	60.4	6430.45	0	1032.45	104.08
20–44	38.4	1686.36	16.47	674.54	76.08
45–64	54.18	872.48	11.59	401.34	82.67
65–74	94.88	2879.30	212.5	1226.99	143.52
75 and older	309.31	8530.80	205.06	3496.50	427.58

**Table 4:** Unadjusted and adjusted Odds Ratios (95% CI) for injury severity in front- and rear-seated occupants aged 18 or older involved in a motor vehicle crash in New York State, CODES.

Covariates	Unadjusted	Predictors of Severe Injury/Death in Multicar Crashes Controlling for Restraint Status	Predictors of Severe Injury/Death in Multicar Crashes without Controlling for Restraint Status
	OR (95%)	OR (95%)	OR (95%)
Driver Gender			
Male	Ref	Ref	Ref
Female	0.952 (0.918, 0.987)	0.762 (0.702, 0.827)	0.766 (0.708, 0.829)
Restraint Status			
Unrestrained	Ref	Ref	N/A
Restrained	0.135 (0.128, 0.142)	0.194 (0.177, 0.212)	N/A
Seating Position			
Front	Ref	Ref	Ref
Rear	1.174 (1.088, 1.266)	0.804 (0.715, 0.904)	1.272 (1.146, 1.412)
Driver Drinking or Drugged			
No	Ref	Ref	Ref
Yes	7.788 (7.356, 8.246)	4.264 (3.890, 4.674)	5.229 (4.805, 5.691)
Speeding			
No	Ref	Ref	Ref
Yes	3.245 (3.096, 3.400)	1.949 (1.805, 2.105)	2.011 (1.867, 2.166)
Ejection Status			
Not ejected	Ref	Ref	Ref
Partially ejected	16.791 (13.923, 20.249)	6.372 (4.607, 8.812)	9.941 (7.397, 13.359)
Fully ejected	42.896 (36.460, 50.469)	13.305 (9.904, 17.875)	31.794 (24.661, 40.990)
Collision Manner			
Rear-end	0.112 (0.103, 0.121)	0.147 (0.131, 0.165)	0.146 (0.131, 0.163)
Overtaking	0.065 (0.058, 0.073)	0.084 (0.072, 0.099)	0.086 (0.074, 0.101)
Left turn	0.179 (0.162, 0.197)	0.226 (0.196, 0.260)	0.223 (0.194, 0.255)
Right turn/angle	0.176 (0.163, 0.191)	0.217 (0.193, 0.244)	0.216 (0.192, 0.241)
Head-on	Ref	Ref	Ref
Sideswipe	0.218 (0.188, 0.252)	0.267 (0.218, 0.328)	0.251 (0.205, 0.307)
Other	0.284 (0.265, 0.305)	0.267 (0.238, 0.300)	0.267 (0.239, 0.299)
Vehicle Year			
<1999	Ref	Ref	Ref
1999 to 2003	0.803 (0.739, 0.872)	0.764 (0.674, 0.866)	0.724 (0.643, 0.816)
2004 to 2008	0.651 (0.601, 0.704)	0.666 (0.592, 0.750)	0.636 (0.569, 0.712)
2009 to 2014	0.504 (0.467, 0.545)	0.543 (0.482, 0.611)	0.514 (0.459, 0.574)
2015 to 2018	0.459 (0.423, 0.499)	0.516 (0.457, 0.582)	0.490 (0.436, 0.550)

## Data Availability

These are state data that are linked under a data use agreement and an IRB that does not provide public data distribution.
